# Patellofemoral pain syndrome (PFPS): a systematic review of anatomy and potential risk factors

**DOI:** 10.1186/1476-5918-7-9

**Published:** 2008-06-26

**Authors:** Gregory R Waryasz, Ann Y McDermott

**Affiliations:** 1 Tufts University School of Medicine, Boston, MA, USA; 2 Department of Nutrition, Brigham and Women's Hospital, Boston, MA, USA; 3 Kinesiology Department, California Polytechnic State University, San Luis Obispo, CA, USA

## Abstract

**Background:**

Patellofemoral Pain Syndrome (PFPS), a common cause of anterior knee pain, is successfully treated in over 2/3 of patients through rehabilitation protocols designed to reduce pain and return function to the individual. Applying preventive medicine strategies, the majority of cases of PFPS may be avoided if a pre-diagnosis can be made by clinician or certified athletic trainer testing the current researched potential risk factors during a Preparticipation Screening Evaluation (PPSE). We provide a detailed and comprehensive review of the soft tissue, arterial system, and innervation to the patellofemoral joint in order to supply the clinician with the knowledge required to assess the anatomy and make recommendations to patients identified as potentially at risk. The purpose of this article is to review knee anatomy and the literature regarding potential risk factors associated with patellofemoral pain syndrome and prehabilitation strategies. A comprehensive review of knee anatomy will present the relationships of arterial collateralization, innervations, and soft tissue alignment to the possible multifactoral mechanism involved in PFPS, while attempting to advocate future use of different treatments aimed at non-soft tissue causes of PFPS.

**Methods:**

A systematic database search of English language PubMed, SportDiscus, Ovid MEDLINE, Web of Science, LexisNexis, and EBM reviews, plus hand searching the reference lists of these retrieved articles was performed to determine possible risk factors for patellofemoral pain syndrome.

**Results:**

Positive potential risk factors identified included: weakness in functional testing; gastrocnemius, hamstring, quadriceps or iliotibial band tightness; generalized ligamentous laxity; deficient hamstring or quadriceps strength; hip musculature weakness; an excessive quadriceps (Q) angle; patellar compression or tilting; and an abnormal VMO/VL reflex timing. An evidence-based medicine model was utilized to report evaluation criteria to determine the at-risk individuals, then a defined prehabilitation program was proposed that begins with a dynamic warm-up followed by stretches, power and multi-joint exercises, and culminates with isolation exercises. The prehabilitation program is performed at lower intensity level ranges and can be conducted 3 days per week in conjunction with general strength training. Based on an objective one repetition maximum (1RM) test which determines the amount an individual can lift in good form through a full range of motion, prehabilitation exercises are performed at 50–60% intensity.

**Conclusion:**

To reduce the likelihood of developing PFPS, any individual, especially those with positive potential risk factors, can perform the proposed prehabilitation program.

## Background

Patellofemoral Pain Syndrome (PFPS) is a term for a variety of pathologies or anatomical abnormalities leading to a type of anterior knee pain [1]. Knowledge of the anatomy of the patellofemoral (PF) joint is essential to developing an understanding of the pathogenesis of PFPS. The symptom of anterior knee pain is associated with the conditions listed in Table 1. Pain may be caused by increased subcondral bone stress attributed to the stress of articulation or from cartilaginous lesions on the patella or distal femur [2-4]. Nearly 10% of all sports injury clinic visits by physically active individuals are attributed to PFPS [5], with more than 2/3 of patients being successfully treated through rehabilitation protocols [6-8].

**Table 1:** Common Pathologies Leading to Anterior Knee Pain (AKP)*

Articular Cartilage Injury	Bone Tumors	Chondromalacia Patellae
Hoffa's Disease	Iliotibial (IT) Band Syndrome	Loose Bodies

Neuromas	Osgood-Schlatter Disease	Osteochondritis Dissecans

Patellar Instability/Subluxation	Patellar Stress Fracture	Patellar Tendinopathy

Patellofemoral Arthritis	Patellofemoral Pain Syndrome	Pes Anserine Bursitis

Plica Synovialis	Prepatellar Bursitis	Previous Surgery

Quadriceps Tendinopathy	Referred Pain from Lumbar Spine or Hip Joint Pathology	Saphenous Neuritis

Sinding-Larsen-Johansson Syndrome	Symptomatic Bipartite Patella	

* Based on research presented by S. Dixit 2007, P. Brukner 2002, R.H. Miller 1998, J.P. Fulkerson 2000, W.E. Prentice 2001, T.A. Peters 2000, R. Khaund 2005, A. Haim 2006

Physical training including sport-specific cardiovascular training, plyometrics, sport cord drills, strength and flexibility training has been found in adolescent female soccer players to significantly reduce lower body injury incidence from 33.7% to 14.3%, allowing athletes to be game-ready [9]. Injuries cannot be prevented entirely, however practitioners can attempt to avoid some types and keep more severe injuries to a minimum [9]. Also, participating with injuries due to insufficient recovery time increases the risk of new injury [10]. Concern over the long term consequences of anterior knee pain in adolescence and young adulthood includes a predisposition to patellofemoral osteoarthritis in later life [11]. The goal of sports medicine should be to keep athletes and patients healthy, pain free, and able to enjoy their sport and physical activity for years to come.

Physical rehabilitation programs to treat anterior knee pain have proven to be a highly effective non-operative option [6-8]. Results have ranged from an 82% success rate in decreasing the severity of symptoms in athletes with chondromalacia patella [7], to an 87% initial success rate for a combination physical therapy and NSAIDs intervention, with 68% maintaining improvements for a mean of 16 months post-rehabilitation [8]. In treating chronic patellofemoral pain syndrome, continuous rehabilitation conducted over seven years had a 67% success rate of complete subjective and functional recovery [6]. These high rehabilitation success rates for anterior knee pain due to either cartilaginous injury or anatomical abnormalities suggest the potential for the prevention of a majority of anterior knee pain using a prehabilitation approach.

PFPS is a condition of both malalignment and muscular dysfunction [1]. Unlike a surgical distal realignment procedure [12,13], rehabilitation exercises can restore PF joint homeostasis although the anatomical malalignment of PFPS may not be corrected [1]. In comparison, prehabilitation aims to optimize function and pain measures before a stressful event [14]. Because the symptoms of anterior knee pain are brought on by overuse stress [15], PFPS is an ideal condition for prehabilitation. It should be noted that the shape and size of the patella and trochlear groove are limiting factors in the outcome of a rehabilitation program [16], and therefore would likely limit the outcome of a prehabilitation program. Based on anatomical, functional, and biomechanical parameters known to occur in symptomatic athletes before overuse resulted in pain, prehabilitation involves a pre-diagnosis in asymptomatic individuals. The Preparticipation Screening Evaluation (PPSE) offers an opportune time to make a pre-diagnosis and initiate a prehabilitation protocol [17].

### Anatomy of the Patellofemoral Region

**Figure 1:**
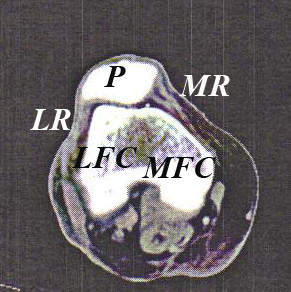
**Cadaver Patellofemoral Computed Tomography Scan**. P- Patella; LR- Lateral Retinaculum; MR- Medial Retinaculum; LFC- Lateral femoral condyle; MFC- Medial femoral condyle.

The patella (Figure 1), the largest sesamoid bone in the human body [18], functions to improve flexion efficiency and to protect the tibiofemoral joint [18]. The combination of the quadriceps tendon, lateral retinaculum, medial retinaculum, and the patella tendon help stabilize the patella [19]. Because the patella is not completely engaged in the patellar groove during the first 0–30 degrees of flexion, instability and the potential for subluxation/dislocation injury increases if patellar stabilizers are weak or malaligned [19].

#### Arterial System

Arterial blood flow to the knee is accomplished by an intricate system of anastomoses between five major arteries: superior medial and lateral, the middle (posterior), and the inferior medial and lateral genicular arteries [20]. An anastomosis occurs between the anterior tibial recurrent artery and the descending genicular arteries [20]. The genicular arteries except for the middle genicular artery make a contribution to the circumpatellar anastomosis [20]. The cirumpatellar anastomosis extends into the superficial and deep structures of the bone, synovium, capsule, retinaculum, and subcutaneous fascia [20]. The arterial supply to the patella arises from the circumpatellar anastomosis [20].

Arising from the popliteal artery, the medial superior genicular artery lies anterior to the semimembranous and semitendinosus muscles, and the lateral superior genicular artery will then anastomose with the descending branch of the lateral collateral femoral artery to supply the vastus lateralis, vastus intermedius, and branches of the femoral nerve [20]. The middle genicular artery passes anterior to the joint line and into the posterior joint capsule to supply the anterior cruciate ligament (ACL) and the posterior cruciate ligament (PCL) [20,21].

The medial inferior and lateral genicular arteries arise from the popliteal artery distal to the posterior joint line and proceed to the deep collateral ligaments [21]. The medial inferior genicular artery provides blood supply to the tibial (medial) collateral ligament, anastomoses with the saphenous branch of the descending genicular branch, and anastomoses with the anterior tibial recurrent artery [20]. The lateral inferior genicular artery forms an anastomosis with the anterior tibial recurrent artery and supplies the fibular (lateral) collateral ligament at the joint line [20].

Gross arterial anatomy is similar between adults and children, however on a microscopic level, there are childhood differences in blood supply to the epiphyseal plate [20]. The pathology of PFPS may be related to decreased pulsatile blood flow in skeletally mature individuals [22]. Tissue ischemia resulting from mechanical forces that reduce genicular arterial flow during passive flexion from 20 to 90 degrees may be a cause or consequence of the pain associated with PFPS [22]. Surgical disruption of the genicular arterial system has not been reported to cause permanent vascular abnormalities to the patella, because the arterial supply appears able to revascularize the patella adequately after a surgical insult during ligamentous reconstruction procedures involving the knee [23]. Such surgical disruption can occur during a lateral retinculum release of the patella [23], a common surgical procedure for the alleviation of PFPS pain. If ischemia is an issue in the pathogenesis of PFPS, an arteriogram or other sophisticated test may detect defects in the collateral flow that could warrant the use surgical or medical revascularization to treat PFPS.

#### Quadriceps Force Vector

**Figure 2:**
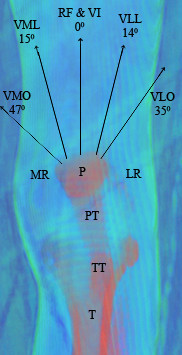
**Quadriceps-Patellar Force Diagram**. VMO- Vastus medialis obliquus; VML- Vastus medialis longus; RF- Rectus femoris; VI- Vastus intermedius; VLL- Vastus lateralis longus; VLO- Vastus lateralis obliquus; P- Patella; TT- Tibial Tubercle; T- Tibia; MR- Medial retinaculum; LR- Lateral retinaculum.

The quadriceps force vector (Figure 2) includes forces from the fiber orientation of the vastus lateralis (VL), vastus intermedius (VI), rectus femoris (RF), and the vastus medialis (VM). The vastus lateralis is composed of two force vector components, the vastus lateralis longus (VLL) and vastus lateralis obliquus (VLO) [19]. The vastus medialis is composed of two force vector components, the vastus medialis longus (VML) and vastus medialis obliquus (VMO) [19]. In the coronal plane, the quadriceps force vector angles are made by the VLO at 35 degrees and the VLL at 14 degrees laterally, by the VI and RF at 0 deg, and medially by VMO at 47 degrees and VML at 15 degrees. Overall the quadriceps force has a posterior pull sagitally to keep the patella in proper articulation with the trochlear groove [19].

The lateral retinaculum is two layers; the superficial oblique retinaculum and a deep transverse retinaculum. The superficial oblique retinaculum is the culmination of the interdigitating of the patellar tendon, the VL group, and the iliotibial (IT) band [16,21,24-26]. The IT band originates from the tensor fascia lata and the gluteus maximus [21], with its attachment on the lateral epicondyle of the femur [12] and Gerdy's Tubercle on the anterior promixal tibia [12,21].

The deep transverse retinaculum consists of three structures; the epicondylopatellar band or lateral patellofemoral ligament, the midportion, and the patellotibial band [26]. The epicondylopatellar band provides superolateral support, the midportion provides lateral support, and the patellotibial band provides inferolateral support to the patella [16,21,24-26]. The midportion originates from the IT band and attaches to the lateral patella [26].

The lateral retinaculum is often released arthroscopically to alleviate its lateral displacement force [27]. To avoid complications, the procedure involves an incision through the superficial oblique retinaculum and deep transverse retinaculum without violating the joint capsule [26].

The medial retinaculum is much thinner than the lateral retinaculum and consists of three ligaments beneath the retinaculum; the medial patellofemoral ligament (MPFL), medial patellomeniscal ligament (MPML), and medial patellotibial ligament (MPTL). The MPFL merges with the VMO forming the primary restrictive mechanism for excessive lateral patella deviation, especially during lower degrees of knee flexion approaching full extension [16], the time when the patella is at greater risk of dislocation/subluxation [19]. Acute lateral patellar dislocation can occur if the MPFL is torn away from the femur or if the VMO muscle is torn from the adductor magnus tendon [28]. There is controversy as to validity of the VMO as being anatomically distinct and functionally separate from the VML [29]. The VM muscle group is both a knee extensor and patellar stabilizer dependent on the task performed [30]. The MPML and MPTL are thought to be less important in PF joint stability than the MPFL [16,19,31,32].

A cadaveric study demonstrated that static medial stability contributions were 50% from the MPFL, 24% from the MPML, 13% from the MPTL, and 13% from the medial retinaculum [33]. Due to the interdigitation with the VMO, the MPFL contributes over 50% against lateral dislocation as it assists to maintain the patella in the trochlear groove during the initial 20–30 degrees of flexion [33].

#### Sensory Receptors

The patellofemoral joint contains a variety of sensory receptors not distinct to this specific joint including: bare nerve endings, Pacinian corpuscles, Ruffini endings, Golgi receptors, and muscle spindles [34]. The major sensory nerves supplying the knee joint are the posterior articular (PAN), lateral articular (LAN), medial articular (MAN), intramuscular, and muscle nerves [34]. PAN is a branch of the tibial nerve that supplies the posterior cruciate ligament, anterior cruciate ligament, posterior oblique ligament, insertion of the annular ligament at the mediolateral menisci, posterior fat pad, posterior capsule, fibular collateral ligament, and the tibial collateral ligament [34]. LAN is a branch of the common peroneal nerve that inconsistently innervates the tibiofibular joint capsule and the lateral knee tissues. MAN is a branch of the saphenous nerve that supplies the anterior and medial capsule, medial meniscus, tibial collateral ligrament, posterior capsule, patellar fat pad, and patellar tendon [34]. The intramuscular and muscle nerves include the golgi tendon organs and muscle spindles supplied by branches of the femoral, obturator, or sciatic nerve depending on the location of the myotome [21,34,35].

The lateral patellar nerve innervates the patella at the lateral anterior border at the 11 o'clock position [36]. The medial patellar nerve innervates the patella at the medial anterior border at the 2 o'clock position. Both the medial and lateral patellar nerves are distal branches of the femoral nerve [36]. The medial based neurovascular bundle is the primary interosseous innervation to the patella [37]. The medial and central portions of the patella are densely interosseosly innervated in comparison to the lateral patella [37].

The innervation to the skin in the anterior region of the knee is from the lateral and anterior cutaneous branches of the femoral nerve and the infrapatellar branch of the saphenous nerve [21,35]. Posterior skin overlying the knee is supplied by the posterior cutaneous nerve, and cutaneous branches of the obturator nerve [21,35].

There is substance-P in the soft tissue supports of the patella including the fat pad, retinaculum, and periosteum, which is evidence for the soft tissue role in anterior knee pain [38]. Substance-P is involved in nociceptive input to the spinal cord and functionis as a vasodilator producing inflammation [38]. Woijtys (1990) observed that substance-P fibers may be denser in the lateral than the medial retinaculum, however the study did not specifically quantify the observation [38]. Substance-P fibers have also been found in the patellar marrow cavity in degenerative knees [38]. Identifying possible nerve defects or increased sensitivity to pain could alter treatment to include corticosteroid injections through regional nerve block techniques that are highly specific to the region of pain.

## Methods

A systematic database search of PubMed, SportDiscus, Ovid MEDLINE, Web of Science, LexisNexis, and EBM reviews, plus hand searching the reference lists of these retrieved articles was performed to determine potential risk factors for patellofemoral pain syndrome. Key words searched were "patellofemoral pain syndrome", "patellofemoral", "anterior knee pain", "chondromalacia patella", "knee", and "patella". Articles were included based upon availability through the Tufts Hirsch Health Science Library and Interlibrary Loan. Selection criteria were based on a subject population with PFPS or a description of anterior knee pain not consistent with other pathologies listed in Table 1 and based on inclusion criteria presented in the article. Articles included were prospective cohorts, case-control, and case series. The articles included were limited to the English language and published between January 1984 and July 2007. Excluded from analysis were articles involving a treatment intervention.

## Results

A total of 24 articles were included in Additional File 1: Review of Potential Patellofemoral Pain Syndrome Risk Factors. There are 3 prospective cohorts [39-41], 17 case-controls [42-58], and 4 case series articles [59-62] included in Additional File 1: Review of Potential Patellofemoral Pain Syndrome Risk Factors. Two articles were included that did not report P values [51,59]. Seven articles were included that did not have a patient population described specifically as PFPS [40,51,55,56,59,63,64], however the articles met the review inclusion criteria presented in the methods section. The articles were included to present the extent of research into the potential risk factors of PFPS that has been performed from January 1984 to July 2007.

### Electromyography (EMG) Measured Neuro-Motor Dysfunction

Using electromyography (EMG) to measure neuro-motor dysfunction in PFPS has been analyzed in 5 studies. All 5 studies have determined that when comparing PFPS subjects to controls, there is significant neuro-motor dysfunction in PFPS. Thomee (1996) demonstrated that the vastus medialis muscle was less active on EMG in PFPS patients, while the rectus femoris was equally active to healthy controls while standing [45]. Cowan (2001) and Cowan (2002) determined that during activities of daily living there was a difference in EMG onset in PFPS compared to controls [42,43]. Witvrouw (2000) found VMO/VL reflex response time to be a significant finding in PFPS [39]. The VMO/VL reflex response time was determined by electromyography unit with skin electrodes over the VL and VMO muscle bellies. Readings were taken using the patellar tendon reflex with the test performed 10 times per knee [39]. The VMO/VL muscles responded faster in the PFPS group compared to the controls [39]. Although not statistically significant, the group noticed that the VMO fired earlier compared to the VL in the control group [39], which would equate to an earlier activation of the medial force vector preventing lateral patella displacement. The authors concluded that an altered VMO/VL response time was a risk factor for PFPS [39]. The authors found no statistical difference when the VL response time was subtracted from the VMO response time (VMO-VL) between the PFPS group and the control group [39]. Crossley (2004) states that although the magnitude differences measured by EMG may be small, but statistically significant detecting these differences may influence treatment [44].

### Foot Abnormalities

The characteristics of genu varum, genu valgum, pes cavus, and pes planus have not been found to contribute to PFPS [39,48] or other related conditions [15,47,65]. Arch index was determined in one study to be significantly lower for only a discriminant analysis of anterior knee pain not specifically classified as PFPS [63]. The arch index was calculated by forming three equal foot sections (forefoot, midfoot, and rearfoot), then dividing the midfoot area by the total footprint area and serves as a marker that the anterior knee pain group had a higher arched foot (cavus) which may produce greater pressures during running on the PF joint [63]. Other literature does suggest an increased risk of running injuries may be due to genu varum, genu valgum, and foot postural abnormalities, including excessive pronation, valgus ankles, and lowered foot arches [12,66-68]. Additional research is needed to clarify the validity of these characteristics as potential risk factors for PFPS.

### Functional Testing

Functional testing may show that PFPS patients have lower strength capacity [39] as demonstrated by decreased vertical jump performance [39,46], anteromedial lunge [49], step-down [49], single-leg press [49], and balance and reach tests [49]. No difference was appreciated between the PFPS patients and controls for Flamingo balance, standing broad jump, bent arm hang, shuttle run, plate tapping, arm pull, leg lifts, sit and reach, sit ups, and maximal oxygen uptake[39]. No research has definitively suggested that PFPS is due to the lower strength capacity or rather a result of lower strength capacity. For this reason, functional testing deficits are a potential risk factor until proven otherwise.

### Gastrocnemius Tightness

Gastrocnemius and soleus tightness reduces the amount of dorsiflexion leading to excessive subtalar joint pronation and tibial internal rotation which will cause femoral internal rotation to increase the Q angle [50]. Therefore, one mechanism to PFPS pathogenesis is by increasing Q angle and increased PF joint stresses [50]. Gastrocnemius tightness was significant in two studies comparing PFPS patients to controls [39,50], but was not significant in another study comparing anterior knee pain subjects to controls [63].

### Generalized Ligamentous/Joint Laxity

Generalized ligamentous laxity is proposed to increase the total patellar mobility which would alter patellar tracking and lead to symptoms [64]. Generalized ligamentous laxity was significantly increased in PFPS patients in two out of three studies. Al-Rawi (1997) found significant generalized ligamentous laxity in chondromalacie patella knees [64]. Witvrouw (2000) was only able to find significance in the thumb-forearm mobility exam, the rest of the exam was not significant [39]. Fairbank (1984) found the relationship between knee pain and generalized ligamentous laxity not to be significant [51].

### Hamstring Strength

The mechanism behind hamstring strength and pathogenesis of PFPS is not well understood, however overall lower body strength is recommended for a runner's exercise program [63] and the hamstrings are involved in power activities such as the vertical jump [69]. Hamstring strength was examined in one study and determined that running athletes with a "syndrome complex" have an 81% absolute strength deficiency at 60 degrees per second and 73% had a deficiency at 240 degrees per second when using a Cybex dynamometer [59]. "Syndrome complex" refers to pain in the anterior aspect of the knee in the soft tissues or around the patellar tendon, pain upon running, mild retropatellar pain upon compression with minimal crepitus, and no clinical evidence of patellar subluxability, chondromalacia, plica, increased Q angle, or increased foot pronation [59]. The data however was not presented as a research article, and no P values were reported [59].

### Hamstring Tightness

Hamstring tightness has been theorized to either cause slight knee flexion during activities or to necessitate higher quadriceps forces to overcome the passive resistance of the hamstring, both of which may increase PF joint reaction forces [50]. Hamstring tightness was evaluated in four articles [39,40,50,59]. Two of the four articles found hamstring tightness in anterior knee pain/PFPS athletes [40,50], one study found no significance [39], and one study stated that 23% of "syndrome complex" athletes had appreciable hamstring tightness [59].

### Hip Musculature Weakness

The iliopsoas muscle, a hip flexor and secondary femoral external rotator, if weak de-stabilizes the pelvis [70,71]. The individual then compensates by developing an anterior pelvic tilt with an internally rotated femur [70,71], the Q angle is then increased, leading to increased PF joint stresses [50]. For the small number of patients who show asymmetrical hip rotation with diminished medial rotation and excessive lateral rotation, a program designed to create a balance in internal and external rotation hip strength is required [72]. A strong VMO with weak hip adductors results in the adductor magnus tendon being drawn to the patella; therefore, strong hip adductors serve as a stable origin for VMO contraction [73].

Two of three studies evaluating hip musculature found weakness [52,53]. Hip abductor strength was determined to be significantly decreased in both studies when comparing PFPS patients to control subjects [52,53]. Piva (2005) found hip external rotation strength and hip abduction strength not be significant [50].

Balanced hip strength is very important for PFPS prevention, as the IT band originates from the lateral hip musculature [12] and the VMO has a relationship to the adductor magnus tendon [28]. A 6-week treatment program designed to improve hip flexion, adduction, and abduction strengths in patients with PFPS (n = 35) led to a combination of improved hip flexion strength with a normalized Ober Test and Thomas Test in 93% of successfully treated PFPS cases defined by a decrease in VAS pain score [74].

### Iliotibial (IT) Band Tightness

IT band tightness through anatomical correlations to the lateral retinaculum and patella will increase the lateral force vector on the patella during flexion to increase the lateral PF joint stresses [54,75]. Iliotibial band tightness was found in PFPS athletes in three articles evaluating the IT band [54,59,60]. An early study Kibler (1987) reported 67% of running athletes with "syndrome complex" had IT band tightness, although there was no P value reported [59]. Piva (2005) reported no tightness in the IT band/tensor fascia lata complex [50].

### Quadriceps Angle (Q-Angle)

A greater Q angle is believed to change the location of contact and pressure in the PF joint, resulting in areas experiencing excessive stresses that are not physiologically manageable [63]. Huberti (1984) using cadaver knees and a special loading fixture found that both an increased and a decreased Q angle increased peak patellofemoral pressures [76]. These increased pressures may predispose an individual to degenerative pathological changes [76]. Increasing the Q angle is associated with increased lateral patellofemoral contact pressures and patellar dislocation, while decreasing the Q angle may not shift the patella medially, but rather increases the medial tibiofemoral contact pressure through increasing the varus orientation of the knee [77]. The effect of Q angle has been examined in a number of studies [39,47,48,55-57,63]. Three studies reported the Q-angle to be significantly increased in PFPS subjects against controls [48,55,57], while four studies reported no difference in Q angle [39,47,56,63].

Haim (2006) reported an abnormal Q angle of greater than 20 degrees was statistically associated with anterior knee pain [48]. Q angle has been believed to differ between males and females, however the slight difference of only 2.3 degrees appears to be related to height rather than pelvic dimensions [78]. Shorter statured individuals appear to have larger Q angles and therefore the slight difference between genders may be attributed to men being taller than women [78].

### Quadriceps Tightness

Witvrouw (2000) states that the decreased quadriceps flexibility existed prior to developing the symptomatic syndrome, and therefore is not necessarily a result of PFPS [39]. Quadriceps tightness may cause high patellofemoral stresses that predispose individuals to developing symptoms [39,79]. The presence of quadriceps tightness was reported in all five studies that evaluated quadriceps tightness [39,40,50,59,63]. While Kibler (1987) reported 61% of "syndrome complex" patients had rectus femoris tightness, no P value was reported [59].

### Quadriceps Weakness

Quadriceps weakness, specifically VMO weakness in comparison to the VL, can lead to lateral displacement of the patella causing the articulating pressure to be on the lateral facet [16,19]. The quadriceps force vector (Figure 2) explains how an imbalance in strength can lead to improper patella alignment as a weak VMO cannot adequately support medial patellar stability [16,19]. A total of six studies evaluated quadriceps weakness on anterior knee pain/PFPS. Two studies reported quadriceps weakness was a non-significant finding [41,57]. Three studies found weakness to be a significant finding [46,58,62]. Kibler (1987) reported that 39% of "syndrome complex" athletes had appreciable quadriceps weakness (no P value reported) [59].

### Patellar Compression/Crepitus

Patellar compression/crepitus was examined in two studies [48,61]. Testing using the patellar tracking test was determined to be 56% sensitive and 55% specific as confirmed by arthroscopy [61]. The patellar tracking test is performed by compressing the patella in the trochlear groove while moving the patella up and down, with pain during the test indicating a positive result for chondromalacia [61]. Haim (2006) found patellofemoral crepitation significantly associated with reduced mobility in PFPS patients [48]. Crepitus alone may be a non-specific finding [75], and therefore may not be useful as a potential risk factor based on the current research.

### Patellar Mediolateral Glide/Mobility

While two studies reported reduced patellar mobility in PFPS patients [48,60], Witvrouw (2000) reported that medial, lateral, and total patellar mobility were greater in PFPS, however the findings were not significant [39]. After assessment of the research of patellar glide/mobility as a risk for PFPS, data appears to be inconclusive at the current time.

### Patellar Tilting

Excess patellar tilting laterally can lead to patellar medial hypomobility resulting in high stresses between the lateral facet of the patella and the lateral trochlea [75]. Excessive tightness of the lateral structures inhibits the patella from reentering the trochlear groove when the pathologic lateral tilt is in excess of 20 degrees when the knee is in extension as measured by CT scan [19]. Haim (2006) reported a positive patellar tilt was significant for PFPS subjects compared to controls with 92% specificity and 43% sensitivity [48].

## Discussion

### Recommendations for Pre-Diagnostic Physical Examination

A number of identifiable and diagnostically accurate risk factors exist that can be determined without radiographic imaging (Table 2) [75]. General visualization of the patellar movement through flexion extension may be helpful in detecting malalignment if there is a "J sign" [80,81] as result of lateral retinacular tightness or medial retinacular weakness. Decreased quadriceps flexibility, specifically rectus femoris tightness, can be assessed by using the Ely Test [82].

**Table 2:** Pre-Diagnostic Evaluation for Patellofemoral Pain Syndrome (PFPS)

**Pre-Diagnostic Criteria**	**Risk Factor Evaluated**	**Instructions**
"J Sign" Visualization [80,81]	Deviation of the patella as the patella engages in the trochlea	• Clinician visualizes the medial deviation during early flexion and the inverted "J" movement of the patella due to tightness of the lateral retinaculum or VMO dysfunction.
		• A positive "J sign" involves lateral deviation of the patella during the terminal extension phase.
Ely Test [82]	Decreased quadriceps flexibility, specifically the rectus femoris muscle	• Athlete lies prone while passive flexion of the athlete's knee is produced for full static ROM with pressure placed on distal 1/3 of lower leg over the tibia.
		• Examiner places other hand over the region of the intertrochanteric line of the anterior femur.
		• If knee flexion causes the athlete's hip on the same side to have a spontaneous flexion contracture, the rectus femoris is deemed to be tight.
		• A comparison should be made between both legs.
Ober Test [74]	Tight Iliotibial (IT) band	• The patient is sidelying with the top leg in knee flexion and the bottom knee extended.
		• The clinician stabilizes the pelvis with one hand and grasps the ankle to guide the lower extremity with knee flexion into hip extension.
		• The upper leg is abducted and extended to keep the thigh in line with the body.
		• A positive test is when the leg does not adduct pain-free medially past the midline, and may indicate a tight IT band.
Thomas Test [74,83-85]	Poor hip flexor flexibility	• The patient lies supine with one leg in hip/knee extension with ankle dorsiflexed.
		• The other leg is in hip/knee flexion with ankle dorsiflexed.
		• The clinician pushes in the region of the tibial tubercle to create greater hip flexion.
		• The patient attempts to gain the greatest (ROM) in hip flexion, while keeping the opposite leg firmly on the ground or examination table.
		• If the iliopsoas is tight, the opposite leg with show initiation of hip flexion through a flexion contracture.
Trendelenburg Test [86]	Weak hip abductors	• Clinician observes the patient standing on one leg. • A positive test is a noticeable drop in the pelvis on the opposite side due to hip instability or weak abductors.
Quadriceps Atrophy [58]	Quadriceps circumference asymmetry	• Clinician determines visually or by using a tape-measurement proximal to the patella.
Altered VMO/VL Response Time [1]	Altered VMO muscle reflex time compared to VL	• Clinician's hands are placed on both the muscle belly of the VMO and the VL while the knee is in extension.
		• Patient is asked to contract the quadriceps group while the clinical feels for a timing difference between VMO and VL contraction.
		• In a normal patient, no timing difference between the contraction of the VMO and VL exists. A positive test is a marked delayed onset of the VMO muscle on palpation.
Vertical Jump/Poor Power Production [39]	Reduction of power production capacity or poor overall lower body force production potential.	• Vertical jump analysis can be performed using a Vertec Device.
		• Parameters are not well defined; however any decrease in vertical jump testing shows decreased power production potential. Care must be taken to perform the test in same test environment conditions as different locations and techniques will change outcome.
Q Angle Measurement [48,55]	Excessive Q angle (greater than 20 degrees)	• Patient stands with the knee in full extension [48].
		• Q angle is formed by the line connecting the ASIS and the center of the patella intersects the line connecting the center of the patella with the middle of the anterior tuberosity.
		• A Q angle measurement in excess of 20 degrees may lead patient to be at a higher risk for PFPS.
Generalized Ligamentous Laxity [39,64,75,103]	Generalized ligamentous laxity	• Either:
		◦ Passive 5^th ^finger digit dorsiflexion beyond 90 degrees.
		◦ Passive apposition of the thumb to the flexor forearm.
		◦ Elbow hyperextension in excess of 10 degrees.
		◦ Knee hyperextension beyond 10 degrees.
		◦ Ability to place the palms of the hands on the floor while maintaining forward flexion of the trunk with knees straight.
		• Having any positive generalized ligamentous laxity characteristics may make the patient higher risk for PFPS.
Patellar Tilt [39,80]	Lateral retinacular tightness	• Lateral retinacular tightness is determined if the lateral patella cannot be raised to horizontal while compressing the medial patella posteriorly.
		• Excessive patellar tilt can be considered positive by the clinician's clinical experience regardless of meeting the exact criteria.

* Based on research presented by S. Dixit 2007, T.F. Tyler 2006, R.H. Miller 1998, B.B. Phillips 1998, A. Haim 2006, E. Witvrouw 2000, C.E. Cook 2007, M.F. Davis 2005, M.L. Ireland 2003, M.J. Callaghan 2004, G.A. Malanga 2006, T.R. Baechle 1995, C.E. Cook 2007, J.P. Fulkerson 2002, M. Fredericson 2006, and J. McConnell 2007.

Abbreviations:

ASIS- anterior superior iliac spine; IT- iliotibial; ROM- range of motion; VL- vastus lateralis; VMO- vastus medialis obliquus

Decreased IT band flexibility is evaluated using the Ober Test [74,79]. Decreased hip flexor flexibility is assessed using the Thomas Test [74,83-85]. Weak hip abductors are evaluated using the Trendelenburg Test [86].

A Q angle measurement in excess of 20 degrees may increase PFPS risk [48], however studies have demonstrated slight differences in Q angle between PFPS and control at lower Q angle values [55,57].

Weak quadriceps or quadriceps atrophy can be determined visually or by using a tape-measure to check for asymmetry between sides. Quadriceps circumference is measured proximal to the patella. The diagnostic parameters have not been well-defined, as the level of atrophy may be minimal [58], however athletes should have near bilateral symmetry. Utilizing the quadriceps atrophy criteria may be left to the opinion of the clinician as to whether there is enough quadriceps tone or if the asymmetry warrants a prehabilitation program prescription.

Altered VMO muscle reflex time compared to VL is assessed by simultaneous VMO and VL palpation during knee extension. In normal patients, no timing difference between the contraction of the VMO and VL exists. In some patients, a marked delayed onset of VMO is evident on palpation [1]. Electromyography using skin electrodes over the VMO and VL could be used to more accurately ascertain the reflex time difference during an elicited patellar tendon reflex using a reflex hammer [39], however this may not be feasible financially for most clinicians.

Decreased vertical jump is assessed by direct measurement, preferably using a Vertec device, however the criteria for jump height, type of jump surface, and specific testing technique are not developed well enough for pre-diagnostic use. Rather, comparison with previous vertical jump testing and a decrease in performance may indicate decreased power production which may translates to an increased risk for developing PFPS [39]. Other functional tests can be performed (see Additional File 1: Review of Potential Patellofemoral Pain Syndrome Risk Factors), however the vertical jump test is a practical way to track athletic progress as a prehabilitation is initiated.

Generalized ligamentous laxity is determined by a variety of tests listed in Table 2. Having any generalized ligamentous laxity characteristics may be a positive indication for PFPS prehabilitation, as studies have showed a significant correlation with generalized ligamentous laxity tests and symptomatic PFPS [39,64,75].

A patellar tilt test can also show lateral retinacular tightness if the lateral patella cannot be raised to horizontal while compressing the medial patella posteriorly [39,80]. There is still the clinician's opinion as to whether or not the athlete has a hypomobile patella even if the criteria are not met. Other pre-diagnostic criteria may be developed or the current criteria altered as larger prospective PFPS studies are conducted and more information is learned. Radiographic measurements are more accurate, but cost effectiveness is a concern.

### Proposed Prehabilitation Intervention

The prehabilitation program is derived from common practices in PFPS rehabilitation and from strength and conditioning trends designed to increase power output, create balanced strength, and reduce overuse injuries associated with symptomatic PFPS (Tables 3 and Additional File 2: Patellofemoral Pain Syndrome (PFPS) Exercises and Prescription Recommendations and Instructions). As with diagnosis, it is important to consider factors in a joint proximal and in a joint distal to the joint of interest. The program consists of a general dynamic warm-up, stretching (13 total), power exercises (1 total), multi-joint exercises (2 total), and isolation exercises (1 total) for each of the defined muscle groups. Isolation exercises described have a major effect on a single muscle group, although have minor effects on other muscle groups as a true isolation is difficult to achieve during exercise. Athletes are encouraged to incorporate this program 3 days per week at rehabilitation intensity levels [light (50% intensity of 1 repetition maximum (1 RM)/heavy (60% intensity of 1 RM)/moderate (55% intensity of 1 RM)], as a supplement to general weight lifting and stretching activities. This intensity is in contrast to "heavy week" training in which the strength and conditioning professional or certified athletic trainer prescribed intensity levels reach up to or near a 1 RM. A variety of general weight lifting programs are outlined by the National Strength and Conditioning Association (NSCA) [69] and should be the primary program of the athlete, with the PFPS prehabilitation program serving as additional, less intense exercises performed to develop symmetrical lower body strength and flexibility. The proposed program is based on a non-linear periodization model and can be made flexible based on athletic training demands. A 1 RM can be determined by a formal maximal weight lift if the athlete demonstrates proper form for a back squat and leg press. The athlete should not experience pain during the 1 RM lift. Other exercises listed in Additional File 2: Patellofemoral Pain Syndrome (PFPS) Exercises and Prescription Recommendations and Instructions require the athlete and fitness practitioner to determine an appropriate weight that pushes the athlete at a targeted rate of perceived exertion (Borg RPE scale) [87]. The 1 RM lift for exercises such as lunges, resistance band training, Romanian Dead Lift (RDL), and box jumps also involve RPE, rather than by actual maximal lift.

**Table 3:** Patellofemoral Pain Syndrome (PFPS) Exercise Prescription Supplement Overview

**Dynamic Warm-Up**	General dynamic warm-up designed by the strength coach or certified athletic trainer. Sample Dynamic Warm-up: High-Knee March, Toe Jogging, Straight Leg Jogging, "Butt-Kickers", High Knee Skip, Side-Shuffles, Forward Lunge-Walk, High Knee Run, Increasing Intensity 65%–100% 10 yd sprints)
**PFPS Stretches**	Thomas Test Stretch/Single Leg Sprinter Stretch
	Ely Test Stretch/Prone Quadriceps Stretch
	Ober Test Stretch
	Supine Active Isolated Stretching (AIS) Gastrocnemius Stretch
	Supine AIS Dorsiflexion Hamstring Stretch
	Supine AIS Plantarflexion Hamstring Stretch
	Long AIS Adductors Stretch
	Four Point Stretch
	Hip Internal Rotation
	Hip External Rotation
	Figure-of-Four Stretch
	Lying Iliotibial (IT) Band Stretch
	Seated Iliotibial (IT) Band Stretch

**Power Exercise**	Box Jumps/Resisted Squat Jumps

**Multi Joint**	40 deg Knee Flexion Squat/60 deg Knee Flexion Leg Press
	Forward Lunge/Step-Ups

**Isolation Hamstrings**	Romanian Dead Lift (RDL)/Back Extension

**Isolation Quadriceps**	Bridges/Closed Kinetic Chain Terminal Knee Extensions

**Isolation Hip Abductors/Adductors**	Manual Resistance (MR) or Thera-band Hip Abductor/Adductor

*Based on research by T.R. Baechle 1995, T.F. Tyler 2006, B.B. Phillips 1998, C.E. Cook 2007, W.E. Prentice 2001, M.B. Roush 2000, K. Crossley 2002, N. Curtis 1995, J. McConnell 2007, and A.L. Mattes 2006.

Once identified as "at risk for developing symptomatic PFPS", the athlete should 1) continually perform the prehabilitation program as long as the athlete wishes to remain physically active, with 2) periodic vertical jump testing to ensure there is no decrease in power production. Due to bone growth changing the lower leg moment of inertia in children [88], the prehabilitation program may not be necessary or appropriate for anterior knee pain prevention in skeletally immature individuals.

Dynamic and static methods of stretching increase both ROM and flexibility for injury prevention, and are both incorporated in rehabilitation [89]. Dynamic stretching is performed during the general dynamic warm-up (Table 3) taught by the certified athletic trainer or strength and conditioning specialist [69]. Dynamic stretching involves controlled movements that gradually increase in speed and range of motion, mimicking the athletic activity to follow so as to increase muscle memory [69]. This is in contrast to ballistic stretching which uses the momentum of a moving limb in a spring-like manner, attempting to force it beyond its normal range of motion [69]. The dynamic warm-up does not include any ballistic stretching, as ballistic stretching has been associated with injury [69]. Static stretching and Active Isolated Stretching (AIS) are performed after the dynamic warm-up [69,90]. Stretching the IT Band, hamstrings, quadriceps, hip adductors, hip abductors, hip external rotators, hip internal rotators, quadriceps, gastrocnemius/soleus, and hip flexors is prescribed for PFPS rehabilitation and therefore is appropriate for prehabilitation [16,74,79,89,91,92].

Power exercises, such as the power clean, snatch, or push jerk, can increase power production if the athlete has the proper instruction and equipment available [69]. The power clean is an Olympic style lift that involves a quick and forceful lift of a bar off the ground to the final position in front of the shoulders through one movement [69]. The snatch is an Olympic style lift that involves quickly and forcefully lifting a bar off the floor to an overhead position in one uninterrupted motion, ending with the elbows in full extension [69]. The push jerk involves rapidly moving the bar from the shoulders to an overhead position using an explosive extension of the hips and knees to accelerate the bar to its final overhead position ending with elbows extended. [69]. Due to the potential for injury from improperly performing Olympic lifting exercises, the box jump and resisted squat jump are included in the supplemental program to improve power output [69]. It is recommended these exercises be performed on an Olympic-style platform with hard-soled shoes, preferably Olympic-style weight lifting shoes.

Rehabilitation protocols have determined that both closed kinetic chain (CKC) and open kinetic chain (OKC) do not create supraphysiologic stresses and are advantageous to the individual with PFPS [28,91,93]. Lower body CKC exercises involve many muscle groups, are typically weight bearing and involve the foot remaining in a fixed position without movement. Examples include the back squat and leg press. CKC exercises are considered superior for athletic purposes [93] based on mimicking functional movements in sport and involving many muscle groups.

In comparison, lower body OKC exercises isolate a specific muscle, are typically non-weight bearing and involve free movement of the foot. Examples include straight leg raises and knee extensions. Both CKC and OKC are included in PFPS rehabilitation programs [91,92,94], with adjusted ranges of motion on traditional exercises (Additional File 2: Patellofemoral Pain Syndrome (PFPS) Exercises and Prescription Recommendations and Instructions).

VMO training is important to improve VL and VMO onset timing differences [95]. Retraining the vasti with eccentric exercises such as squats has been noted to improve PFPS rehabilitation outcomes [96]. VMO muscle isolation has been difficult to prove possible without the use of electrode stimulation [97], however it is generally believed that VMO activity is greater with the hip in external rotation [97]. Hip external rotators have been determined to be weaker in PFPS patients as diagnosed by the single-leg squat test, therefore loading in external rotation is beneficial [13], even if there is no added benefit to the VMO. Many protocols have successfully emphasized the use of a 10 o'clock and 2 o'clock (between 30–45 degrees) position of femoral external rotation [98].

The hip's external/internal rotation, flexion/extension, and abduction/adduction groups need to be both stretched and strengthened [74,79,89,91,99,100]. Isolation exercises for these groups, as well as multi-joint exercises that focus on eccentric loading and isometric contraction, are important [99].

Historically, patellar taping has been advocated in treating PFPS patients to increase VMO activity and decrease VL activity [101]. In asymptomatic individuals the data are limited and conflicting, with patellar taping noted to be effective [102] or detrimental [101], therefore the prehabilitation program does not promote use of patellar taping until supported by additional research.

## Summary

In skeletally mature patients, anatomical abnormalities may be pre-diagnosed during the Preparticipation Screening Evaluation (PPSE) by using the evidence-based criteria for potential PFPS risk factors. The clinician with a proper knowledge of the neurovascular, bony, and muscular anatomy has the knowledge to appropriately assess malalignment of the PF joint and therefore perform a screening physical examination for PFPS based on potential risk factors. The anatomy section also serves as a reference point to 1) explain exactly how anatomical deviations can potentiate PFPS pathogenesis and 2) stimulate thought about other possible therapies, including addressing vascular insufficiency and neuropathic pain. In an effort to prevent the onset of debilitating knee pain, a positive finding in any pre-diagnostic category or asymptomatic PFPS that concerns the physician results in prophylactic treatment prescribing a prehabilitation exercise protocol based upon proven, successful rehabilitation techniques that create balanced lower body strength, increased flexibility, and increased power production. As more research is conducted on PFPS risk factors and potential risk factors, the pre-diagnostic criteria should be updated and changes made to the supplemental prehabilitation program. By conducting prospective cohort studies in healthy individuals, research could determine whether the risk factors listed in this article serve to initiate or contribute to PFPS, or rather result from PFPS development. The proposed supplemental prehabilitation program offers a safe and effective means to develop balanced lower body strength and flexibility in any individual and should be considered along with a more intense strength training program as necessary for injury prevention and performance enhancement. The proposed program is to be understood as an example of a possible program, other programs can be made that accomplish a similar task of attempting to prevent PFPS. Practitioners are encouraged to alter the program to make it more specific to the athlete and utilize available resources.

## Abbreviations

AIS: Active isolated stretch; Deg: degree(s); MR: Manual resistance; RDL: Romanian dead lift; Yd: Yard

## Competing interests

The authors declare that they have no competing interests.

## Authors' contributions

GRW conceptualized the idea of the pre-diagnostic criteria, prehabilitation exercise protocol, and was the principal author of the manuscript, AYM was responsible for significant reviewing and assistance with the writing and final formatting of the article.

## Supplementary Material

Additional file 1 Review of Potential Patellofemoral Pain Syndrome Risk Factors. Comprehensive table of the articles discussed in the results section of the manuscript that review the potential risk factors for Patellofemoral Pain Syndrome. Click here for file

Additional file 2 Patellofemoral Pain Syndrome (PFPS) Exercises and Prescription Recommendations and Instructions. Table of recommendations and instructions for exercises and stretches suggested to possibly prevent Patellofemoral Pain Syndrome. Click here for file
