# Supplementary material for: Patellofemoral pain syndrome (PFPS): a systematic review of anatomy and potential risk factors
Source: Dyn Med. 2008 Jun 26;7:9. doi: 10.1186/1476-5918-7-9 (PMC2443365; doi:10.1186/1476-5918-7-9)
Supplement: Additional file 1 — Review of Potential Patellofemoral Pain Syndrome Risk Factors. Comprehensive table of the articles discussed in the results section of the manuscript that review the potential risk factors for Patellofemoral Pain Syndrome. [file 1476-5918-7-9-S1.doc]

**Additional File 1: Review of Potential Patellofemoral Pain Syndrome Risk Factors**

| **Test** | **Source** | **Comments** | **Significance** |
| --- | --- | --- | --- |
| **Electromyography (EMG) Measured Neuro-Motor Dysfunction** | Cowan et al (2001)  [43] | 33 PFPS and 33 asymptomatic controls  Case-Control | Significant in PFPS population for EMG onset of VL prior to VMO in both step up and step down phases of stair-stepping activity (P<0.05).  There was no difference in the onset of VMO and VL activity in the asymptomatic controls (P>0.05). |
|  | Cowan et al (2002)  [42] | 37 PFPS and 37 asymptomatic controls  Rise task is standing on toes.  Rock task is standing on heels.  Case-Control | Significant difference in PFPS compared to asymptomatic controls in EMG VL- VMO onset timing difference in rock (P<0.001) and rise (P<0.01).  Significant difference in PFPS group for EMG VL onset compared to VMO onset in rock (P<0.005) and rise (P<0.005).  There was no difference in the VMO and VL activity in asymptomatic controls in rock ( P=0.31) and rise (P=0.33) |
|  | Crossley et al (2004)  [44] | 48 PFPS and 18 controls  Case-Control | Significant stance-phase knee flexion angle is lower in individuals with PFPS compared to controls (P<0.05).  Significant mean onset of VMO EEG activity was delayed relative to that of the VL (P<0.05). |
|  | Thomee et al (1996)  [45] | 11 PFPS and 9 control subjects  Case-Control | Significant (P<0.05) difference between PFPS and controls in vastus medialis EMG activity during standing. PFPS has less activity.  No difference in EMG activity for sitting or standing rectus femoris or sitting vastus medialis (P>0.05).  Average pain during sitting and standing was significantly higher (P<0.0001) using the visual analogue scale (VAS) pain scores. |
|  | Witvrouw et al (2000)  [39] | 24 PFPS and 258 control subjects.  Prospective Cohort | Significant for faster response in VMO (P=0.02) and VL (P=0.006) in the PFPS group compared to controls.  Not significant for difference between response of the VMO-VL (P=0.26) when compared to controls. |
| **Foot Abnormalities** | Duffey et al (2000)  [63] | 99 anterior knee pain and 70 controls subjects.  Case-Control | Significant (P=0.05) for lower arch index (cavus/ higher arches) for combined discriminant analysis only. |
|  | Haim et al (2006)  [48] | 61 PFPS and 25 control subjects. Genu varum, genu valgum, pes cavus, and pes planus investigated.  Case-Control | Not significant for :  Genu varum (P=0.12)  Genu valgum (P=0.21)  Pes cavus (P=1.00)  Pes planus (P=0.15) |
|  | Thomee et al (1995)  [47] | 40 PFPS and 20 control subjects. Leg-heel alignment measurements taken.  Case-Control | Not significant for lower leg anatomical differences (no P value reported). |
|  | Witvrouw et al (2000)  [39] | 24 PFPS and 258 control subjects.  Prospective Cohort | Not significant for genu varum/valgum (P=0.96). |
| **Functional Testing** | Loudon et al (2002)  [49] | 29 subjects with PFPS and 11 controls. The 5 functional tests were anteromedial lunge, step-down, single-leg press, bilateral squat, balance and reach.  Case-Control | PFPS subjects demonstrated decreased performance in anteromedial lunge, step-down, single-leg press, balance and reach when compared to healthy controls (P<0.05). Intrarater interclass correlation coefficient: 0.79-0.94. |
|  | Thomee et al (1995)  [46] | 40 PFPS and 20 control subjects.  Case-Control | Significant for decreased unilateral counter movement drop vertical jump in PFPS subjects (P<0.05) |
|  | Witvrouw et al (2000)  [39] | 24 PFPS and 258 control subjects. Tested Flamingo balance, vertical jump, standing broad jump, bent arm hang, shuttle run, plate tapping, arm pull, leg lifts, sit and reach, sit ups, and maximal O2 uptake.  Prospective Cohort | Significant for a decreased vertical jump (P=0.01) in PFPS subjects.  Not significant for a difference between PFPS and control subjects for Flamingo balance (P=0.021), standing broad jump (P=0.41), bent arm hang (P=0.6), shuttle run (P=0.8), plate tapping (P=0.79), arm pull (P= 0.17), leg lifts (P=0.49), sit and reach (P=0.25), sit ups (P=0.41), and maximal oxygen uptake (P=0.96). |
| **Gastrocnemius Tightness** | Duffey et al (2000)  [63] | 99 anterior knee pain and 70 control subjects.  Case-Control | Not significant (P>0.05) for a difference in anterior knee pain subjects compared to control subjects. |
|  | Piva et al (2005)  [50] | 30 PFPS and 30 control subjects.  Case-Control | Significant (P<0.001) for both increased gastrocnemius and soleus tightness in PFPS subjects. |
|  | Witvrouw et al (2000)  [39] | 24 PFPS and 258 control subjects.  Prospective Cohort | Significant (P=0.038) for increased gastrocnemius tightness. |
| **Generalized Ligamentous/Joint Laxity** | al-Rawi et al (1997)  [64] | 115 chondromalacia patellas and 110 control subjects.  Case-Control | Significant (P<0.001) for increased joint laxity in knees with chondromalacia compared to controls. |
|  | Fairbank et al (1984)  [51] | 136 knee pain and 310 control subjects.  Case-Control | Not significant (no P value reported) for increased joint laxity in knee pain subjects. |
|  | Witvrouw et al (2000)  [39] | 24 PFPS and 258 control subjects.  Prospective Cohort | Significant for increased thumb-forearm mobility in PFPS subjects compared to controls (P=0.01).  Not significant for increased extension of the little forefinger (P=0.058), shoulder mobility (P=0.06), elbow extension (P=0.41), and knee extension (P=0.37) in PFPS subjects compared to controls. |
| **Hamstring Strength** | Kibler (1987)  [59] | 76 running athletes with “syndrome complex”.  Case series | 81% of “syndrome complex” subjects had an absolute strength deficiency at 60deg per second and 73% had a deficiency at 240 degrees per second (No P value reported) |
| **Hamstring Tightness** | Kibler (1987)  [59] | 76 running athletes with “syndrome complex”.  Case series | 23 % of “syndrome complex” subjects had tightness (no P value reported). |
|  | Piva et al (2005)  [50] | 30 PFPS and 30 control subjects.  Case-Control | Significant for hamstring tightness in the PFPS subjects (P<0.001). |
|  | Smith et al (1991)  [40] | 14 anterior knee pain and 32 control subjects.  Prospective Cohort | Significant for hamstring tightness in the anterior knee pain subjects (P<0.01). |
|  | Witvrouw et al (2000)  [39] | 24 PFPS and 258 control subjects.  Prospective Cohort | Not significant for hamstring tightness in the PFPS subjects (P=0.442). |
| **Hip Musculature Weakness** | Cichanowski et al (2007)  [52] | 13 PFPS and 13 matched control subjects. PFPS subjects were only included if unilateral pain. Injured leg was compared to non-injured leg in PFPS group.  Case-Control | Significant for hip abduction (P=0.003) and external rotation weakness (P=0.049) when comparing injured to uninjured knee. Not significant for hip flexion (P=0.466), extension (P=0.563), adduction (P=0.650), and internal rotation (P=0.111).  Significant for hip flexion (P=0.033), extension (P=0.029), abduction (P=0.01), internal rotation (P=0.049), and external rotation (P=0.033) weakness. Not significant for hip adduction (P=0.087) |
|  | Ireland et al (2003)  [53] | 15 PFPS and 15 control subjects.  Case-Control | Significant for 26% less strength in hip abductor in the PFPS subjects (P<0.001). |
|  | Piva et al (2005)  [50] | 30 PFPS and 30 control subjects.  Case-Control | Not significant for hip external rotation strength weakness (% of body mass) (P=0.218) or hip abduction strength weakness (% of body mass) (P=0.016). |
| **Iliotibial Band Tightness** | Kibler (1987)  [59] | 76 running athletes with “syndrome complex”.  Case series | 67% of “syndrome complex” subjects had IT band tightness (No P value reported). |
|  | Piva et al (2005)  [50] | 30 PFPS and 30 control subjects.  Case-Control | Not significant for iliotibial band/ tensor fascia lata complex length (P=0.102) difference. |
|  | Puniello (1993)  [60] | 17 PFPS subjects.  Case series | 12 of 17 PFPS patients exhibited IT band tightness (P<0.005). |
|  | Winslow et al (1995)  [54] | 14 PFPS and 34 control subjects.  Case-Control | Significant for IT band tightness in PFPS subjects(P<0.01) |
| **Quadriceps-Angle**  **(Q-Angle)** | Aglietti et al (1983)  [55] | 150 healthy knees and 90 chondromalacia patella knees.  Case-Control | Significant for increased Q angle in chondromalacia patella knees (P<0.001). |
|  | Caylor et al (1993)  [56] | 50 Anterior Knee Pain subjects and 20 healthy subjects.  Case-Control | Not significant for difference in Q angle in anterior knee pain compared to healthy control subjects (P=0.07). |
|  | Duffey et al (2000)  [63] | 99 anterior knee pain and 70 controls.  Case-Control | Not significant for difference in Q angle in anterior knee pain compared to healthy control subjects (P>0.05). |
|  | Haim et al (2006)  [48] | 61 PFPS and 25 control subjects. 32 PFPS had Q angle greater than 20 degrees. No controls had Q angle greater than 20 degrees.  Case-Control | Significant for PFPS if the Q angle is greater than 20 degrees (P<0.001). |
|  | Messier et al (1991)  [57] | 16 PFPS and 20 controls.  Case-Control | Significant for increased Q angle in PFPS subjects between PFPS and control subjects (P<0.01). |
|  | Thomee et al (1995)  [47] | 40 PFPS and 20 control subjects.  Case-Control | Not significant for Q angle difference (P not reported). |
|  | Witvrouw et al (2000)  [39] | 24 PFPS and 258 control subjects.  Prospective Cohort | Not significant for Q angle difference between PFPS and control subjects (P=0.394). |
| **Quadriceps Tightness** | Duffey et al (2000)  [63] | 99 anterior knee pain and 70 controls.  Case-Control | Significant for increased quadriceps tightness in anterior knee pain subjects (P=0.022). |
|  | Kibler (1987)  [59] | 76 running athletes with “syndrome complex”.  Case series | 61% of “syndrome complex” subjects have tightness in the rectus femoris (No P value reported). |
|  | Piva et al (2005)  [50] | 30 PFPS and 30 control subjects.  Case-Control | Significant for quadriceps tightness in the PFPS subjects (P<0.001). |
|  | Smith et al (1991)  [40] | 14 anterior knee pain and 32 control subjects.  Case-Control | Significant for quadriceps tightness in the PFPS subjects (P<0.01). |
|  | Witvrouw et al (2000)  [39] | 24 PFPS and 258 control subjects.  Prospective- Cohort | Significant for quadriceps tightness in PFPS subjects (P=0.028). |
| **Quadriceps Weakness** | Bennett et al (1986)  [62] | 130 anterior knee pain subjects.  Case series | 41/130 subjects demonstrated decreased torque production during eccentric exercise between 30-60 degrees of knee flexion (P<0.05). |
|  | Callaghan et al (2004)  [58] | 57 PFPS and 10 control patients.  Case-Control | Significant for quadriceps weakness in PFPS subjects (P=0.002). |
|  | Kibler (1987)  [59] | 76 running athletes with “syndrome complex”.  Case series | 39% of “syndrome complex” subjects have quadriceps weakness (No P value reported). |
|  | Messier et al (1991)  [57] | 16 PFPS and 20 control subjects.  Case-Control | Not significant for quadriceps weakness in PFPS (P>0.05). |
|  | Milgrom et al (1991)  [41] | 77 PFPS knees of 390 military recruits.  Prospective Cohort | Not significant for quadriceps weakness in PFPS (P>0.05). Increased isometric strength of the quadriceps tested at 85 degrees of knee flexion was increased in patellofemoral pain caused by overactivity (P=0.05). |
|  | Thomee et al (1995)  [46] | 40 PFPS and 20 control subjects.  Case-Control | Significant (P<0.01) for eccentric quadriceps weakness in PFPS.  Not significant (P>0.05)) for concentric and isometric quadriceps weakness in PFPS. |
| **Patellar Compression/Crepitus** | Niskanen et al (2001)  [61] | 85 anterior knee pain knees. Data confirmed by arthroscopy confirmation.  Case series | Patellar Tracking test:  Sensitivity 56%, Specificity 55% for detecting chondromalacia in anterior knee pain. |
|  | Haim et al (2006)  [48] | 61 PFPS and 25 control subjects. 43 PFPS and 2 control subjects with PF crepitations.  Case-Control | Significant crepitations for PFPS subjects (P<0.001). |
| **Patellar Mediolateral Glide/Mobility** | Haim et al (2006)  [48] | 61 PFPS and 25 control subjects. Patellar glide measured as a percentage of patellar width.  Case-Control | Significant (P=0.018) reduced mobility in PFPS patients. |
|  | Puniello (1993)  [60] | 17 PFPS subjects.  Case series | 14 or 17 PFPS patients exhibited hypomobility of the medial glide (P<0.005). |
|  | Witvrouw et al (2000)  [39] | 24 PFPS and 258 control subjects.  Prospective Cohort | Not significant (P=0.06), but medial, lateral, and total patellar mobility was greater in PFPS group. |
| **Patellar Tilting** | Haim et al (2006)  [48] | 61 PFPS and 25 control subjects.  Case-Control | Significant (P=0.002) for lateral tilting. Specificity 92% Sensitivity 43% |
