# Supplementary material for: Patellofemoral pain syndrome (PFPS): a systematic review of anatomy and potential risk factors
Source: Dyn Med. 2008 Jun 26;7:9. doi: 10.1186/1476-5918-7-9 (PMC2443365; doi:10.1186/1476-5918-7-9)
Supplement: Additional file 2 — Patellofemoral Pain Syndrome (PFPS) Exercises and Prescription Recommendations and Instructions. Table of recommendations and instructions for exercises and stretches suggested to possibly prevent Patellofemoral Pain Syndrome. [file 1476-5918-7-9-S2.doc]

**Additional File 2: Patellofemoral Pain Syndrome (PFPS) Exercises and Prescription Recommendations and Instructions**

| **Exercise Type** | **Exercise Name** | **Instructions** | **Light:**  **50% intensity** | | **Heavy:**  **60% intensity** | | **Moderate:**  **55% intensity** | |
| --- | --- | --- | --- | --- | --- | --- | --- | --- |
|  |  |  | **Sets** | **Reps or time** | **Sets** | **Reps or time** | **Sets** | **Reps or time** |
| ***Power Exercises*** |  |  |  |  |  |  |  |  |
|  | **Resisted Squat Jump** | •A barbell or weighted stick is placed in Olympic-Style back squat position.  •Starting position is hips externally rotated 30 to 45 deg (10 o’clock and 2 o’clock) and feet apart so that the medial malleolus is in line with the greater trochanter of the femur.  •During the descent to ¼ squat position before the jump, care is taken to maintain external rotation of the hip so that the knees do not endure a valgus stress and limit flexion of the hip to attempt to keep the vertebral column close to perpendicular to the platform.  •The athlete jumps straight up keeping the bar positioned in Olympic-Style back squat position and lands with the same starting foot position. | 3 | 8 | 4 | 8 | 4 | 6 |
|  | **Box Jump** | •Starting position is hips externally rotated 30 to 45 deg (10 o’clock and 2 o’clock) and feet apart so that the medial malleolus is in line with the greater trochanter of the femur. The knee and hip are flexed to enter the “scoop” phase of the power clean. The athlete stands within 12 inches from the padded box.  •The athlete descends to jump. As the athlete jumps, there is a “cycling of the heels” to allow the athlete to land on the box in an athletic position with the knees flexed less than 90 deg stopping the center of gravity (as if performing a power clean correctly) and hips remaining in external rotation.  •The athlete then steps down slowly from the box and sets up for another repetition.  •The height of the box is too high if, during the landing phase on the box, the athlete finishes with knee flexion greater than 90 degrees. | 3 | 8 | 4 | 8 | 4 | 6 |
| ***Multi-Joint Exercises*** |  |  |  |  |  |  |  |  |
|  | **40^0^ Knee Flexion Squats** | •A barbell or weighted stick is placed in Olympic-Style back squat position.  •Starting position is hips externally rotated 30 to 45 deg (10 o’clock and 2 o’clock) and feet apart so that the medial malleolus is in line with the greater trochanter of the femur.  •During the descent of the squat, care is taken to maintain external rotation of the hip so that the knees do not endure a valgus stress and limit flexion of the hip to attempt to keep the vertebral column close to perpendicular to the platform.  •The descent phase of the squat is to 1/3 to 1/2 of a full parallel Olympic Style Back Squat (around 40 degrees of knee flexion). Care should be taken to keep the entire sole of the foot in contact with the platform during the entire exercise  •The ascent phase of the squat is to between 0 to 5 degrees of knee flexion with care taken to keep the hips externally rotated and the vertebral column as close to perpendicular to the platform. The knee should not be in hyperextension at the culmination of the repetition. | 3 | 8 | 4 | 8 | 4 | 6 |
|  | **60^0^ Knee Flexion Leg Press** | •Starting position is hips externally rotated 30 to 45 deg (10 o’clock and 2 o’clock) and feet apart so that the medial malleolus is in line with the greater trochanter of the femur  •During the descent of the leg press, care is taken to maintain external rotation of the hip so that the knees do not endure a valgus stress  •The descent phase of the leg press is to 60 deg of knee flexion.  •The ascent phase of the leg press is to between 0 to 5 deg of knee flexion to keep from entering hyperextension. Care should be taken to keep the medial epicondyle of the knee aligned with the medial malleolus by maintaining external rotation of the hips and keeping the entire sole of the foot in contact with the platform during the entire exercise. | 3 | 8 | 4 | 8 | 4 | 6 |
|  | **Forward Lunge** | •Starting position is hips externally rotated 30 to 45 deg (10 o’clock and 2 o’clock) and feet less than 6 inches apart.  • The athlete takes a step forward maintaining the hip external rotation as the torso maintains perpendicular to the platform. The trailing foot is maintained in the starting position.  •The descent of the lunge involves flexion of the knee and flexion of the hip. The flexion of the knee should keep the knee in final position to stay a few inches posterior to the Hallux, but anterior to the metatarsal joint line. The trailing leg undergoes knee flexion with the final position for the knee being about 1-2 inches above the floor.  •The athlete ascends from this position to the start position maintaining the hip external rotation during the full ascent.  •The exercise is repeated with the trailing leg becoming the lead leg. | 3 | 8 | 4 | 8 | 4 | 6 |
|  | **Forward Step-Ups** | •Starting position is hips externally rotated 30 to 45 deg (10 o’clock and 2 o’clock) and feet apart so that the medial malleolus is in line with the greater trochanter of the femur.  •The athlete stands 12 inches from the box (note: height of box for rehab is 6-8 inch box and for training can be a 12-18 inch box)  •The athlete steps onto the box and plants foot maintaining external rotation of the hip. The athlete then pushes off the box to initiate a “cycling” of the trailing leg to finish with the trailing leg in a position where the thigh is parallel to the floor, the lower leg is perpendicular to the ground, and the ankle is dorsiflexed.  •The trailing leg is then brought back to the starting position on the ground.  •The lead leg is then brought back to the starting position on the ground.  •The exercise is repeated with the trailing leg becoming the lead leg. | 3 | 8 | 4 | 8 | 4 | 6 |
| ***Isolation***  ***Exercises*** |  |  |  |  |  |  |  |  |
| **Hamstrings** |  |  |  |  |  |  |  |  |
|  | **Romanian Dead Lift (RDL)** | •Starting position is hips externally rotated 30 to 45 deg (10 o’clock and 2 o’clock) and feet apart so that the medial malleolus is in line with the greater trochanter of the femur. The knee and hip are flexed to enter the “scoop” phase of the power clean.  •The bar is descended from the proximal 1/3 of anterior thigh to the tibial tubercle as the hip flexes and the knee extends with the feet remaining firmly planted on the platform. The athlete’s back should remain flat without excessive lumbar curvature as flexion of the hip and not the vertebral column is performed. The bar should travel within 1-2 inches of the leg at all times during the descent phase.  •The bar then ascends from the tibial tubercle to the starting point, the proximal 1/3 of the anterior thigh using the hamstrings to pull as the knee flexes and the hips extend in simultaneously. | 3 | 8 | 4 | 8 | 4 | 6 |
|  | **Back Extension** | •Athlete uses back extension apparatus with ventral surface of hands placed behind the region of the C1 vertebrae, but not touching.  •Athlete starts at 0 deg of hip flexion and maintains isometrically contracted abdominals.  •Athlete descends to 45 deg of hip flexion.  •Athlete ascends to 0 deg of hip flexion utilizing the hamstrings and gluteus maximus as prime movers. | 3 | 8 | 4 | 8 | 4 | 6 |
| **Quadriceps** |  |  |  |  |  |  |  |  |
|  | **Bridges** | •Athlete lies supine on two benches separated so the scapula and calcaneus are in contact with separate benches.  •A light weight can be centrally placed over the pelvis resting on the bony prominence of the Anterior Superior Iliac Spine (ASIS) bilaterally.  •The rectus abdominus is contracted isometrically to support the lumbar spine.  •One leg is externally rotated at the hip to between 30 and 45 deg of flexion (10 o’clock and 2 o’clock).  •The same leg is raised using hip flexion, knee extension, and dorsiflexion of the ankle for a period of time.  •The raised leg is lowered to rest on the bench.  •The alternate leg is then raised in the same position for the same amount of time. | 3 | 30 s (15 s each leg) | 4 | 50 s (25 s each leg) | 4 | 30 s (15 s each leg) |
|  | **Closed Kinetic Chain Terminal Knee Extension** | •Starting position is hips externally rotated 30 to 45 deg (10 o’clock and 2 o’clock) and feet apart so that the medial malleolus is in line with the greater trochanter of the femur.  •One end of physical therapy band is securely attached to a fixed piece of equipment or wall mount, while the other end is securely attached around the knee joint.  •The knee is flexed to 30 deg and then returned to between 0 to 5 deg flexion. | 3 | 8 | 4 | 8 | 4 | 6 |
| **Hip Abductors/ Hip Adductors** |  |  |  |  |  |  |  |  |
|  | **Manual Resistance (MR) or Thera-band Hip Abductor/ Adductor** | •Athlete lies in decubitus position if manual resistance or stands if using physical therapy band.  •Athlete performs set of resisted hip abduction through full ROM with pressure applied from a partner or physical therapy band at the region of the lateral epicondyle.  •Athlete performs a set of resisted hip adduction through full ROM with pressure applied from a partner or physical therapy band at the region of the medial epicondyle. | 3 | 8 | 4 | 8 | 4 | 6 |
| ***Stretches*** |  |  |  |  |  |  |  |  |
|  | **Thomas Test Stretch/ Single Leg Sprinter Stretch** | •Athlete lies supine with one leg in hip/knee extension with ankle dorsiflexed. The other leg is in hip/knee flexion with ankle dorsiflexed.  •The partner or athlete pushes/pulls in the region of the tibial tubercle to create greater hip flexion.  •The athlete attempts gain greatest ROM in hip flexion, while keeping the opposite leg firmly on the ground. A partner can apply a force in region of the tibial plateau of the flexed leg towards the chest, while applying a downward force to the distal 1/3 of the anterior thigh to keep hip flexion from occurring. | 1 | 30 s | 1 | 60 s | 1 | 45 s |
|  | **Ely Test Stretch/ Prone Quadriceps Stretch** | •Athlete lies prone while a passive flexion of the athlete’s knee is produced for full static ROM with pressure placed on distal 1/3 of lower leg over the tibia. | 1 | 30 s | 1 | 60 s | 1 | 45 s |
|  | **Ober Test Stretch** | •Athlete lies on side holding the bottom leg around the knee in hip and knee flexion. The top leg is in hip extension with knee flexion.  •The partner stabilizes the hip with one hand, while applying a hip extension and adduction force from the quadriceps tendon region of the top leg with the other hand | 1 | 30 s | 1 | 60 s | 1 | 45 s |
|  | **Supine AIS Gastrocnemius Stretch** | •The athlete sits supine with hips flexed and knees in extension.  •The tibialis anterior muscle in contracted causing active dorsiflexion to maximum dynamic ROM.  •Either a partner or a stretching band is then used to assist in increasing the ROM of the ankle, while maintaining the knee in extension. This point of maximal dorsiflexion is held for 2 seconds.  •The ankle is then plantarflexed passively.  •Another repetition is performed with the same ankle.  •At the culmination of the set, the alternate ankle should be stretched. | 1 | 6 | 1 | 10 | 1 | 8 |
|  | **Supine AIS Dorsiflexion Hamstring Stretch** | •The athlete lies supine with hips and knees in extension. Ankle is dorsiflexed. The athlete should bilaterally keep the hips on the floor throughout the stretch and keep the other leg in hip and knee extension with dorsiflexion on the floor.  •The athlete contracts the quadriceps muscle group to maximally dynamically flex the hip.  •Either a partner or a stretching band is then used to assist in increasing the flexion angle of the hip while maintaining the knee in extension and the ankle in dorsiflexion. This point of maximal hip flexion is held for 2 seconds.  •The knee and hip are then flexed passively.  •Another repetition is performed with the same leg.  •At the culmination of the set, the alternate leg should be stretched. | 1 | 6 | 1 | 10 | 1 | 8 |
|  | **Supine AIS Plantarflexion Hamstring Stretch** | •The athlete lies supine with hips and knees in extension. Ankle is plantarflexed. The athlete should bilaterally keep the hips on the floor throughout the stretch and keep the other leg in hip and knee extension with dorsiflexion on the floor.  •The athlete contracts the quadriceps muscle group to maximally dynamically flex the hip.  •Either a partner or a stretching band is then used to assist in increasing the flexion angle of the hip while maintaining the knee in extension and the ankle in dorsiflexion. This point of maximal hip flexion is held for 2 seconds.  •The knee and hip are then flexed passively.  •Another repetition is performed with the same leg.  •At the culmination of the set, the alternate leg should be stretched. | 1 | 6 | 1 | 10 | 1 | 8 |
|  | **Long AIS Adductors Stretch** | •The athlete lies supine with flexed and adducted hip, extended knee, and dorsiflexed ankle.  •The athlete contracts the hip abductor group to attempt to touch the lateral malleolus to the ground, while stretching the hip adductor group.  •Maximum dynamic ROM is met and then the athlete can assist using pressure from the hands in the region of the adductor tubercle of the femur.  •In full ROM the stretch is held for 2 seconds.  •The athlete then returns to start position and performs another repetition. | 1 | 6 | 1 | 10 | 1 | 8 |
|  | **Four Point Stretch** | •The prone athlete starts in 90 degrees of knee flexion, dorsiflexion, and maximal hip abduction. The four points of contact of the lower extremity with the ground are the left and right medial malleolus and the left and right medial epicondyle of the knee. Upper body support comes from the ulnar side of each hand and medial epicondyle of the elbow being in contact with the floor.  •The athlete maintains a flat back by isometrically contracting the abdominal musculature. | 1 | 30 s | 1 | 60 s | 1 | 45 s |
|  | **Hip Internal Rotation** | •Athlete lies supine with knees in flexion and internal rotation of the hips.  •The athlete spreads the feet laterally and attempts to touch the medial epicondyles of the knee.  •If the medial epicondyles of the knees touch, the feet can be repositioned laterally to increase the internal ROM. | 1 | 30 s | 1 | 60 s | 1 | 45 s |
|  | **Hip External Rotation** | •The athlete lies supine; one knee is flexed and rotated externally.  •The athlete’s hand is placed above the medial malleolus pulling superiorly. The athlete’s other hand is placed over the knee pushing inferiorly.  •Maximum ROM of hip external rotation is met and the stretch is held. | 1 | 30 s | 1 | 60 s | 1 | 45 s |
|  | **Figure-of-Four Stretch** | •The athlete lies supine.  •The athlete flexes and externally rotates the hip of the leg to be stretched.  •The athlete flexes the contra lateral leg and places the leg to be stretched in between it and the torso.  •The athlete reaches through and grabs the shin of the contra lateral leg and pulls in towards the chest, thus stretching the externally rotated leg. | 1 | 30 s | 1 | 60 s | 1 | 45 s |
|  | **Lying IT Band Stretch** | •The athlete lies supine with one leg and knee in extension with dorsiflexion. The other leg is in adducted in hip and knee flexion.  •The athlete pushes on the lateral epicondyle of knee of the leg to be stretched.  •The stretch is held in maximum static ROM. | 1 | 30 s | 1 | 60 s | 1 | 45 s |
|  | **Seated IT Band Stretch** | •The athlete sits will hip flexion, knee extension, and ankle dorsiflexion with no lumbar curvature.  •The leg to be stretched is adducted with a flexed knee over the extended leg.  •The athlete pulls medially on the lateral condyle of the knee with the contra lateral hand. The athlete rotates his neck and torso to the side of the stretched leg keeping the hips on the ground. | 1 | 30 s | 1 | 60 s | 1 | 45 s |

*Based on research by T.R. Baechle 1995, T.F. Tyler 2006, B.B. Phillips 1998, C.E. Cook 2007, W.E. Prentice 2001, M.B. Roush 2000, K. Crossley 2002, N. Curtis 1995, J. McConnell 2007, A.L. Mattes 2006, and original interpretation and modification of exercises by GRW to optimize specific muscle power, flexibility, and strength.

Abbreviations:

AIS- Active isolated stretch; Deg- degree(s); IT- Iliotibial, MR- Manual resistance; RDL- Romanian dead lift; ROM- Range of motion
